# A Statistical Method for Observing Personal Diploid Methylomes and Transcriptomes with Single-Molecule Real-Time Sequencing

**DOI:** 10.3390/genes9090460

**Published:** 2018-09-19

**Authors:** Yuta Suzuki, Yunhao Wang, Kin Fai Au, Shinichi Morishita

**Affiliations:** 1 Department of Computational Biology and Medical Sciences, Graduate School of Frontier Sciences, The University of Tokyo, Tokyo 277-8561, Japan; yuta_suzuki@edu.k.u-tokyo.ac.jp; 2 Department of Internal Medicine, University of Iowa, Iowa City, IA 52242, USA; yunhaowang@126.com; 3 Department of Biomedical Informatics, Ohio State University, Columbus, OH 43210, USA

**Keywords:** statistical methods, DNA methylation, gene expression, single molecule real-time sequencing, allele-specific analysis

## Abstract

We address the problem of observing personal diploid methylomes, CpG methylome pairs of homologous chromosomes that are distinguishable with respect to phased heterozygous variants (PHVs), which is challenging due to scarcity of PHVs in personal genomes. Single molecule real-time (SMRT) sequencing is promising as it outputs long reads with CpG methylation information, but a serious concern is whether reliable PHVs are available in erroneous SMRT reads with an error rate of ∼15%. To overcome the issue, we propose a statistical model that reduces the error rate of phasing CpG site to 1%, thereby calling CpG hypomethylation in each haplotype with >90% precision and sensitivity. Using our statistical model, we examined *GNAS* complex locus known for a combination of maternally, paternally, or biallelically expressed isoforms, and observed allele-specific methylation pattern almost perfectly reflecting their respective allele-specific expression status, demonstrating the merit of elucidating comprehensive personal diploid methylomes and transcriptomes.

## 1. Introduction

DNA methylation plays important regulatory roles in a wide range of biological processes, including differentiation, transposon repression, and cancer progression [1,2,3]. Several technological advances now enable us to evaluate genome-wide DNA methylation [4] at the resolution of a single base-pair [5]. Furthermore, single-cell biology can now be applied to epigenetics, allowing methylation to be measured at the single-cell level. This creates a unique research frontier [6,7,8]. Despite such advances in methodology, the detection of allele-specific methylation (ASM) [9], in which only one of two homologous chromosomes is methylated in a specific region, remains challenging.

To distinguish two homologous chromosomes directly, several studies have explicitly utilized heterozygous variants, as such variants define the differences between two homologous chromosomes. One approach involves a two-step experiment [10,11]. In the first step, DNA fragments containing methylated alleles were enriched using a methylation-sensitive restriction enzyme or by methylated DNA immunoprecipitation. In the second step, sequence variants in the library were quantified using a single nucleotide polymorphism (SNP) array or DNA sequencing. Variants associated with methylation might thus be over-represented when compared with an appropriate negative control. This approach is relatively cost-effective and comprehensive, but the resolution is limited by the distribution of the relevant restriction enzyme cleavage sites, which are far sparser than CpG sites.

Another approach exploits heterozygous variants within bisulfite-treated sequencing reads [12,13]. To assign a read to one of two alleles, the read must contain at least one informative (i.e., heterozygous) variant in addition to the CpG site. However, we will show below that this condition is rarely satisfied when short bisulfite-treated reads are used; bisulfite cleaves DNA into fragments less than 500 bp long [14], with maximum read lengths of 1500 bp [15]. For example, Kuleshov et al. performed haplotyping of a genome using a read cloud containing long-range information and performed short-read bisulfite sequencing to survey ASM in a genome-wide manner; however, ASM was only partially observed [16].

It is difficult to observe comprehensive ASM for a given individual genome because of the lack of sufficient heterozygous variants available in short reads around the CpG sites. Consequently, the current genome-wide overview of ASM is an average mixture of observations for many individuals in a population.

In the present work, we hypothesize that long reads are necessary to directly observe genome-wide ASM of most CpG sites in an individual genome. We developed an alternative method that allows evaluation of regions of intermediate methylation status. We used kinetic information obtained by PacBio (Pacific Biosciences, Menlo Park, California, US) sequencing to call regional CpG methylations as reported previously [17]. We term the allele-specific methylome data obtained using phased long reads as the personal diploid methylome, as these data are comprehensive genome-wide ASM data obtained from a single individual based on personal haplotype information.

Previous studies have revealed the prevalence of allele-specific expression (ASE) in humans and demonstrated a link between ASM and ASE [18,19]. We incorporated transcriptome data obtained using long reads (as in “Iso-seq” studies using PacBio long reads [20]) and short reads to confirm that some of the ASM statuses we detected are consistent with their transcriptional activity, including their ASE statuses, i.e., personal diploid transcriptomes.

## 2. Materials and Methods

### 2.1. Data Source

DNA sequencing data and phased variant information for HG002 were obtained from a file transfer protocol (FTP) repository of the GIAB (Genome in a Bottle) consortium [21]. The original cell lines are available (Coriell GM24385). DNA/RNA sequencing data and assembled haplotigs (haplotype A and B) for AK1 were obtained from a public repository (Accession No. PRJNA298944) [22]. Both samples were lymphoblastoid cell lines.

### 2.2. Assignment of Reads to Each Haplotype for AK1 and HG002

**Figure 1:**
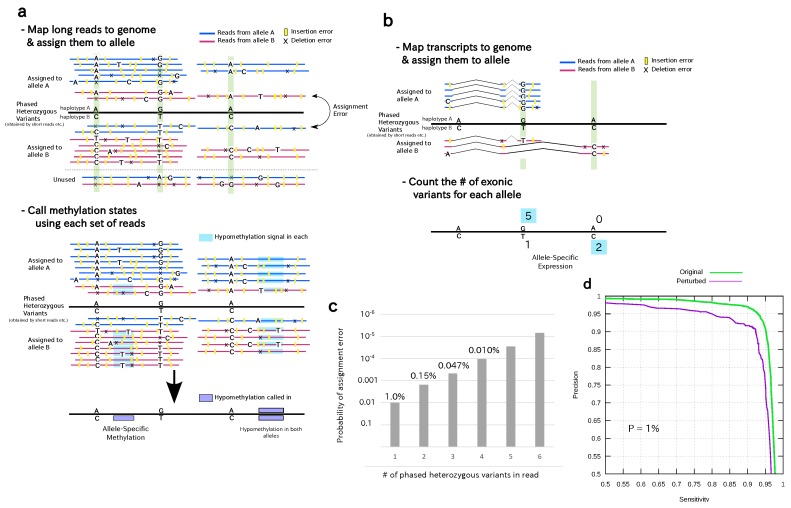

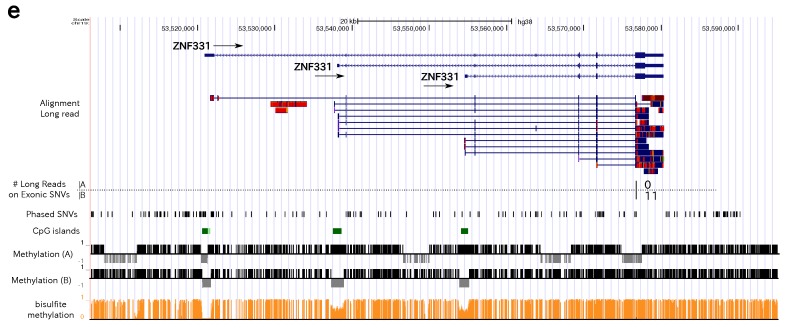
Outline of the proposed method to detect allele-specific methylation (ASM). (**a**) In our method, we assume that haplotype information for the genome is available, i.e., phased heterozygous variants (PHVs) exist (horizontal line in the middle with letters indicating PHVs), which serve as sites of interest (sites shaded in green) for the allele assignment process. Available heterozygous variants found in reads are indicated by letters (A, C, G, or T) at the shaded sites. Other mismatches and insertions/deletions (indels) (shown as letters and yellow blocks) in reads can be assumed to be sequencing errors and therefore ignored. After assigning PacBio reads to either allele (haplotype) by PHVs on the reads, the methylation status of each allele is predicted using (average) kinetics data obtained in the PacBio sequencing process (shaded in blue). Of note, if ASM is not present in the region, wrong assignment of reads does not affect the accuracy of the methylation call; (**b**) Outline of the detection of allele-specific expression (ASE). Only exonic PHVs (two of three in this figure) can be used to distinguish two alleles. Next, ASE can be detected as an imbalance of alleles observed in reads. (**c**) The probability of allele assignment error depends on the number of available PHVs in the reads (*x*-axis). The probability was calculated using an equation presented in the text and is shown in logarithmic scale on the *y*-axis; (**d**) prediction performance (sensitivity and precision) of the method for assessing the perturbed inter-pulse duration (IPD) ratio (purple line). For comparison, typical performance statistics for the original IPD are shown (green line). “P=1%” indicates that the IPD was perturbed to simulate 1% read assignment error, as described in the text; (**e**) Example of a region exhibiting ASM in diploid methylomes and transcriptomes. On the right: two CpG islands (CGIs) are shown in the middle; one allele (labeled A) is methylated and the other (B) unmethylated. Each CGI overlaps with the promoter regions of distinct isoforms of a known imprinted gene *ZNF331*. Bisulfite sequencing data in the bottom track exhibited intermediate-level methylation for the two CGIs showing ASM. From top to bottom, the panel shows the following features: gene structure, alignments of long RNA-seq (Iso-seq) reads, RNA-seq read counts for two alleles, which indicates ASE, sites of PHVs available in this personal genome (black marks), which were used to determine the allelic origins of the sequencing reads, annotated CGIs (green rectangles), methylation levels of the CpG sites of two alleles that were predicted using single-molecule real-time (SMRT) reads (respective black and gray bars towards positive and negative indicate methylated and unmethylated, respectively), and publicly available data on methylation levels via bisulfite sequencing (orange bars).

For AK1 data, contigs of haplotypes A and B were separately aligned to human hg38 reference genome by BWA aligner (version 0.7.15) [23] with the “mem” mode. The phased heterozygous single nucleotide variants (SNVs) were called by comparing the haplotype A and haplotype B genomes. For both samples, we mapped the genomic reads to the reference using BLASR with default options set in SMRT Analysis 2.3.0 (Pacific Biosciences, Menlo Park, California, US) (Figure 1a). Then, if the read contained matches to phased heterozygous variants (PHVs), we counted the number of PHVs supporting each allele. Assignment of the allele for each read was determined by majority voting, and reads were excluded from further analysis if they contained none of the PHVs, or if the voting was tied. In total, 24,181,074 reads (210,782 Mb) from the AK1 dataset were aligned to hg38. The average length of the mapped reads was 8717 bp. Of the reads, 13,857,752 (139,467 Mb) contained at least one match to a PHV, and a haplotype label was assigned. Although these reads constituted 57.3% of all mapped reads in terms of read number, they contained 66.2% of the mapped bases. More bases were retained because longer reads were more likely to contain matches to the PHV. In other words, reads with no matches were likely to be shorter, therefore affecting a relatively small number of bases. Consequently, the average length of reads assigned to a haplotype was 10,064 bp, 115% that of the average length in the original dataset.

For the HG002 dataset, the phased variants calculated using the linked-read technology were available [24]. Starting from 23,031,407 reads (168,051 Mb) aligned to hg38, 13,676,974 reads (111,543 Mb) were assigned a haplotype label. Thus, we retained 59.4% of all mapped reads and 66.4% of all mapped bases. The average read length of reads assigned to a haplotype was 8156 bp, 112% that of the average length of the original dataset, which was 7296 bp.

### 2.3. Generating the Diploid Methylomes

We examined the single-molecule real-time (SMRT) read sets of both alleles separately and called the regional methylation status of genome-wide CpG sites using the kinetic information inherent in reads, as described previously [17]. To determine the cause of read assignment errors in the ASM detection pipeline and how they affect the accuracy of final ASM calls, we assumed that Inter-pulse duration (*IPD*) ratio statistics around the SNV are perturbed by

(1) ΔIPD=random(-1,1)×P×(IPD-1.0),

where random(-1,1) is sampled from the uniform distribution over [-1,1] and P=1%. The third factor, (IPD-1.0), captures a typical scale of IPD deviation. The second factor (P) represents probability of read assignment error, which was estimated to be 1% (Section 3).Then, the perturbation will be largest (fully realized) when two alleles are in completely contrastive methylation state (100%/0% of the molecules methylated), which corresponds to the extreme case where the first factor =1 or -1. In general, we cannot know underlying methylation states in each allele, and we must utilize random distribution to mimic the magnitude of perturbation realized. Moreover, we cannot assume a specific distribution for *IPD* perturbation, thus we decided to use uniform distribution indifferently.

### 2.4. Calculating the Distribution of Phased Heterozygous Variants with Respect to CpG Islands or Exons

CpG island (CGI) annotation was retrieved from the UCSC Genome Browser. For both PHVs and common SNPs, the distance from the CGI was calculated as the genomic distance from the center of the CGI. To calculate the distribution of PHVs and common SNPs with respect to exons, a gene model (GENCODE ver. 24) was intersected with each feature.

### 2.5. Allele-Specific Expression Analysis of AK1

Short-read (Illumina, San Diego, California, US) RNA-seq data was aligned to hg38 reference genome by Hisat2 aligner (version 2.0.0-beta) [25] with the default parameter. For long-read (PacBio) Iso-seq data, CCS reads were extracted from raw h5 files by SMRT Analysis software (version 2.3.0) with the parameter “–minFullPasses 0 –minPredictedAccuracy 70”; then, CCS reads were aligned to hg38 reference genome by GMAP aligner (version 2016-08-16) [26] with the default parameter. IDP-ASE software [19] was used to perform ASE analysis at the gene and isoform levels by short-read RNA-seq data and long-read Iso-Seq data.

### 2.6. Identifying CpG Islands with Allele-Specific Methylation

Of the 26,866 CGIs in the entire genome, we examined 20,140 with at least 30 CpGs to focus on the more functional CGIs. Of these, in the HG002 dataset, 5063 were not covered by long reads after allelic origin assignment, partly because they are relatively distant (4016 are separated by ≥5000 bp) from their nearest PHV. We required that all CGIs be covered by a sufficient number (≥16.0× for each haploid) of long reads to reduce the false discovery rate [17]. A total of 7093 CGIs met these criteria. Similarly, we analyzed the AK1 dataset and determined that 7322 (of 20,140) CGIs were not covered by any read, but 10,087 of the remaining 12,818 CGIs had sufficient coverage (≥16.0×). We then examined the methylation status of each CpG site using our methylation detection algorithm AgIn [17]. Specifically, we calculated the methylation level of the CGI as the (unweighted) average of the methylation status, presented as 0 or 1 (unmethylated or methylated, respectively), within the CGI.After identifying ASM CGIs, each CGI was associated with a gene(s) by manual inspection on a genome browser, and the closest gene (transcript) was recorded.

## 3. Results

### 3.1. A Statistical Model for Accurate Read Assignment

The first step towards ASM detection using long reads is read assignment (Figure 1a), i.e., to assign each read to an original allele based on variants contained in the read. This is a nontrivial task considering that long reads have a higher error rate. While the consensus accuracy of long reads can be sufficiently good for detecting small genomic variants, here we are faced with a raw read error rate as we assign each single read to an allele one by one. It is difficult to call heterozygous variants (found in ∼0.1% of all genomic positions) using only long reads with an error rate of, for example, ∼15%.

We instead utilized highly accurate haplotype PHVs determined by short read sequencing to achieve reliable read assignment. By this design, we were able to ignore discordance between the reference genome and long reads on non-variant sites, which effectively corrected most of the errors in the long reads. Another technique employed to improve read assignment was to use only SNVs and to ignore insertions and deletions (indels), which constitute a dominant fraction of the errors in PacBio long reads, thereby reducing the relevant error rate. This observation has been utilized by several authors to handle erroneous long reads, in diploid-aware de novo assembly [27] and in hybrid error-correction procedure [28], but no explicit modelling has been done for the error probability of long reads phasing. As we will see, even after omitting indels from the analysis, sufficient SNVs are available for read assignment to haplotypes. PacBio read assignment for transcripts (Iso-seq reads) can be similarly performed using heterozygous variants found in exons (Figure 1b).

We can now calculate the expected error rate of the read assignment. Suppose there is only one heterozygous SNV within a read. According to the typical error profile, here we assumed we would observe erroneous insertion (10%), deletion (5%), or substitution to any of the wrong bases (3%) at each position in the PacBio read [29].

Then, read assignment error occurs if and only if the base at an SNV site is substituted by one of the other three bases that incidentally supports the other allele with a probability of 1% (3% substitution rate divided by three). Thus, the error rate of read assignment is expected to be approximately 1% for a read with one SNV. Other types of sequencing errors, indels, and substitutions to bases other than one supporting the wrong allele do not cause read assignment error because such reads are not assigned to either allele.

With more than one heterozygous SNV within a read, the accuracy of read assignment improves drastically. If there are two heterozygous SNVs, then there are two cases where read assignment should occur: First, simultaneous substitution errors resulting in the bases of the wrong allele occur at both sites, giving two variants supporting the wrong allele. The probability of such an event is $1\%\times 1\%=1.0\times 10^{-4}$; Second, one of the SNVs is lost by deletion (5%) or another substitution (2%) in the read, and specific substitution occurs at the other site. In this case, only one variant, which supports the wrong allele, is observed in the read. The probability for this event is 21×(5%+2%)×1%=1.4×10-3. As these two cases are mutually exclusive, the read assignment error rate is $1.0\times 10^{-4}+1.4\times 10^{-3}=1.5\times 10^{-3}=0.15\%$ for a two-SNV read.

Generally, read assignment error occurs when the number of SNVs supporting the wrong allele is greater than that of SNVs supporting the correct allele. If the read has *N* SNVs, and *k* SNVs are missed, then the read would be assigned to the wrong allele if more than half (denoted by *l* below) of the remaining (N-k) SNVs support the wrong allele. Thus, the probability of assignment error for a read with *N* SNVs is

(2) ∑k=0N-1Nk(5%+2%)k{∑l=⌈(N-k)/2⌉N-kN-kl(1%)l},

which is approximately 0.15%,0.047%, and 0.010%, when N=2,3, and 4, respectively. Therefore, the overall assignment error rate decreases exponentially with the number of available heterozygous SNVs (Figure 1c).

Due to the limited availability of a ground truth dataset for personal diploid methylomes, it is difficult to quantitatively assess the accuracy of ASM detection using experimental data. Therefore, we approximated the accuracy by considering major potential sources of errors. To examine the effect of read assignment errors, we added random perturbation, which was proportional to the frequency of read assignment errors, to the IPD ratio of every position independently. Using this setting, the predictive performance of the method was almost unchanged (both the sensitivity and precision were >90%; Figure 1d), presumably because random errors were averaged out in our read assignment. While our analysis simplifies the real situation, it conveys why sequencing errors would not severely affect the accuracy of the method contrary to the impression. Therefore, we conclude that the major source of inaccuracy of the method is methylation detection itself, which is guaranteed to be highly accurate (>93%) [17].

### 3.2. Generating Diploid Methylomes and Transcriptomes for the AK1 and HG002 Datasets

To demonstrate our method of calling ASM, we used two independent datasets: AK1 (Asian Korean) [22] and HG002 (Ashkenazi Trio son) [30], according to the procedure illustrated in Figure 1a (see the details in Methods). The resulting set of methylation calls is a personal diploid methylome, as it comprises two methylomes, each representing one haploid. For the AK1 dataset, RNA-seq data obtained using long and short reads were available and thus used to support the hypothesis that the differential methylation between two alleles that we detected was associated with differential transcription activity. RNA-seq data were mapped to the genome, and then the number of reads supporting transcription from each allele was recorded (Figure 1b), building a pair of transcriptomes in two homologous chromosomes, which we call personal diploid transcriptomes.

Figure 1e shows an example of ASM detected using our method in the genomic region encoding an imprinted gene, *ZNF331*. There are three CGIs in this region, and each CGI corresponds to a promoter region in distinct isoforms of the *ZNF331* gene. While the CGI to the left in the panel was unmethylated for both alleles, the other two CGIs (in the middle and to the right) showed ASM, and our methylation calls informed us that the same allele (i.e., allele B) was unmethylated. Of note, a publicly available methylation-level annotation[31] from a different sample by bisulfite sequencing suggested that these two CGIs are in an intermediate methylation status. The alignment of long-read transcripts and read counts at the exonic PHV supported that the corresponding two isoforms were transcribed exclusively from allele B. Thus, these results suggest that the detected ASM is correlated with the transcriptional activity of the genes. We will cover other examples in later sections to generalize this observation.

### 3.3. Distribution of Phased Heterozygous Variants in Two Personal Genomes

**Figure 2:**
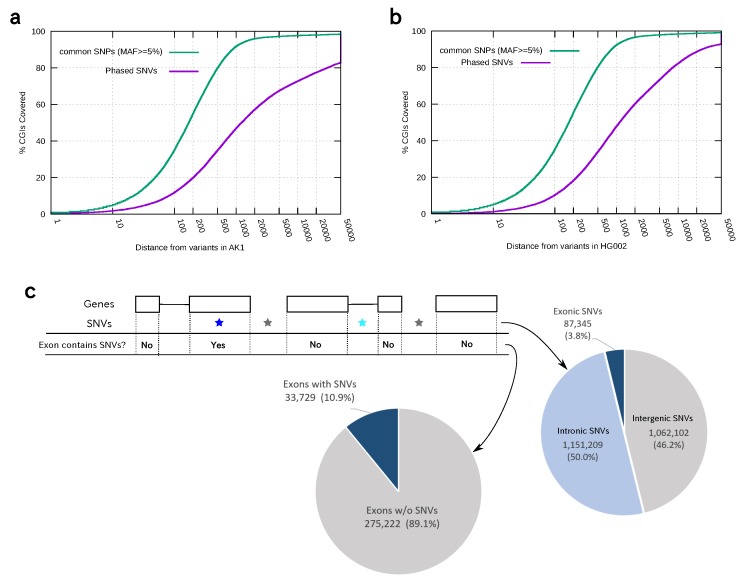

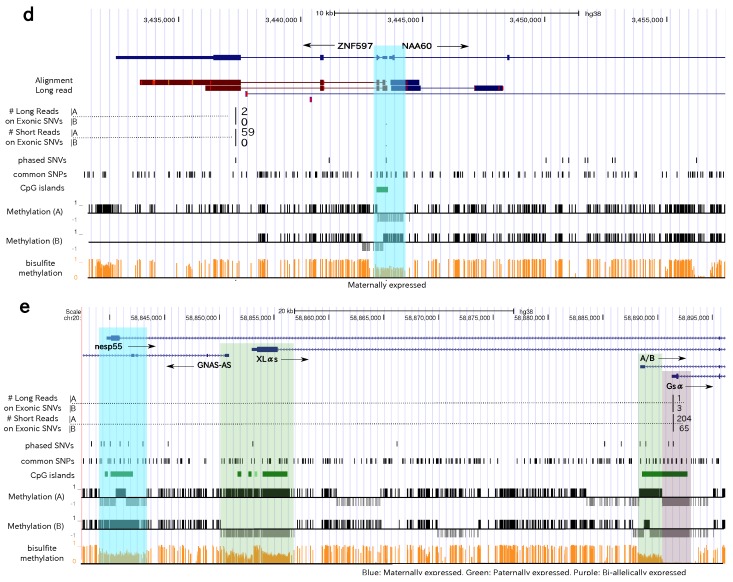
(**a**,**b**) Proportions of CGIs located within a distance in the *x*-axis from the nearest genomic feature, common single nucleotide polymorphisms (SNPs) (green) and heterozygous single nucleotide variants (hetSNVs, or PHVs) (purple), in each genome of (**a**) AK1 and (**b**) HG002. Common SNPs and PHVs distributed differently in both personal genomes. PHVs were essential in determining the proportions of CGIs; (**c**) distribution of PHVs with respect to exons. The left pie chart shows the proportion of exons containing PHVs for which the ASE status can be assessed directly. The right pie chart shows the ratios of PHVs in exonic (blue), intronic (pale blue), or intergenic (gray) regions, thus classifying PHVs into three categories; (**d**) example showing personal diploid methylomes and transcriptomes in the AK1 genome. The CGI in the bidirectional promoter region (area shaded in blue) of the *ZNF597* and *NAA60* genes showed ASM. The RNA-seq reads (both long and short) support that transcription was only derived from allele A, which is the unmethylated allele in the region; (**e**) Personal diploid methylomes around the *GNAS* complex locus in the AK1 genome. The four regions are colored to show their known transcriptional pattern: maternally expressed (blue), paternally expressed (green), or expressed from both alleles (purple). Correspondingly, these regions shaded with different colors exhibited distinct methylation patterns. Of note, the ASM regions exhibited an intermediate level of methylation according to bisulfite sequencing (bottom). RNA-seq reads suggested the expression of Gsα from both alleles.

We next examined how the possibility of assigning reads and CpGs into alleles would be limited by the distribution of PHVs in personal genomes. Specifically, given a read of length *l* bp containing a CpG site, the allelic origin of the read can be determined only when the nearest PHV is located within *l* bp from that site, and both the CpG site and PHV are covered by the same single read. Therefore, to assess this approach, we calculated the proportions of CpGs or CGIs located within a specific distance from the nearest PHV (Figure 2a,b). Of note, these factors depend on the distribution of PHVs available in a given sample and thus can be quite different among individual samples.

One may use the set of SNPs for which the minor allele frequencies are ≥5% in at least 1 of 26 major populations of dbSNPs [32]. As we observed that 83.0% of CpG sites are located within 500 bp from common SNPs (Figure 2a), it would be possible for relatively short reads to determine the allelic origin of themselves if these common SNPs are heterozygously present in an individual genome. However, the conditions posed by the real distribution of PHVs are more severe. Indeed, in the AK1 (HG002) dataset, at most 11.3% (12.3%), 33.7% (37.5%) and 46.1% (51.1%) of CpGs were apparent using read lengths of 100, 500 and 1000 bp, respectively, whereas 72.2% (81.3%) of CpGs were apparent using a read length of 8000 bp (Figure 2a,b, purple line). Therefore, longer reads are essential to detect ASM in real-world situations.

When we try to detect ASE using these PHVs, the only variants we can rely on are those appearing in the RNA-seq reads, i.e., exonic variants. Thus, the situation becomes even more difficult for ASE analysis. For the AK1 dataset, 46.2% of the PHVs were found in intergenic regions and 50.0% in intronic regions (Figure 2c). Thus, only the remaining 3.8% of variants were present in exons to call ASE in this individual. Similarly, 89.1% of ∼310 k exons do not contain such variants, limiting the possibility of determining the expressing allele. While the low abundance of PHVs within exons is largely explained by the fact that exons constitute only a small fraction of the genome, the density of PHVs is also smaller in exons, presumably because of selective pressure on the coding sequences. On average, there were 0.68 PHVs within exons (0.79 PHVs within introns) per 1 kbp.

### 3.4. Allele-Specific Methylation of CpG Islands and Allele-Specific Expresion

Given the fact that exonic phased variants are found only in a small number of transcripts, genome-wide observation of ASM would provide alternative information about the transcriptional activity of individual genomes. To prove this concept, we generated diploid methylomes for the AK1 dataset and compared it with ASE analysis of RNA-seq data for the same dataset (Figure 2d,e).

Shown in the middle of the first panel, a CGI is located in the promoter region of *ZNF597* (Figure 2d). We detected ASM around this CGI, and allele A was unmethylated upstream of the transcription start site (TSS) of *ZNF597*; thus, we predicted that the gene is expressed exclusively from allele A. Consistent with the prediction, we found two long reads and 59 short reads, supporting transcription from allele A, while no reads supported the other allele, B. This identification of ASE was based on an exonic SNV within the last exon, which was the only exonic SNV present in this region, highlighting the sparseness of PHVs in exons.

The second example region is the *GNAS* complex locus, where several isoforms of the *GNAS* gene show ASE (Figure 2e) [33]. Specifically, while *Gsα* (shown at the right end) is expressed from both alleles, the *A/B* transcripts, *XLαs* is paternally expressed (allele B in Figure 2e), and *NESP55* is maternally expressed (allele A). We confirmed that *Gsα* was expressed from both alleles, as the exon specific to the isoform contained a PHV, and both alternative alleles were observed in the RNA-seq reads. For the other isoforms, although we could not directly observe their allelic expression patterns due to the lack of phased variants in exonic regions, the CGIs located in the promoter regions of each isoform showed an ASM pattern consistent with the expected expression pattern; the two CGI regions in the promoters of the *A/B* transcripts, *XLαs*, and *GNAS-AS* were allele-specifically methylated on the same allele, *B*, and the CGI at the promoter of *NESP55* was methylated on the other allele, *A*. Thus, we could predict the expected expression pattern for this locus via its methylation pattern.

These examples demonstrate that the detected ASM status of CGIs can reflect the expression status of the corresponding genes/isoforms. Therefore, such ASM of CGIs would provide useful information, especially when ASE is difficult to detect because of the absence of phased variant sites within exons.

### 3.5. Statistics of Allele-Specific Methylation CpG Islands

**Figure 3:**
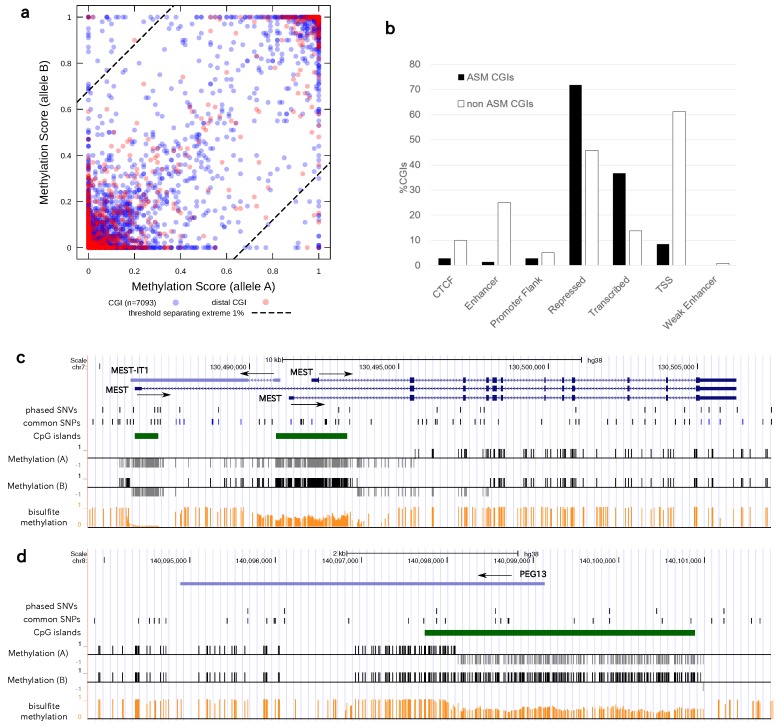
(**a**) Summary of the methylation scores (for each allele) for CGIs in personal diploid methylomes in HG002. Each CGI is shown as a circle. On the two opposite corners (top left and bottom right), CGIs with the top 1% absolute differences in methylation levels between the two alleles were provisionally classified as ASM CGIs. Red circles: The corresponding CGIs were separated from the nearest PHV by 1000 bp or more. Blue circles: Separation <1000 bp; (**b**) distribution of each type of CGI, ASM (black bar) or non-ASM (white bar), with respect to the functional annotation of genomic regions; (**c**) example showing personal diploid methylomes in the *MEST* gene-coding region of the HG002 (Ashkenazim Trio Son) genome. Although the upstream CGI (with 66 CpG sites) was unmethylated in both alleles, the larger downstream CGI (with 184 CpG sites) exhibited ASM. The CGIs corresponded to the promoter regions of different isoforms of the genes; (**d**) another example of ASM around an imprinted gene *PEG13*, paternally expressed gene 13.

Applying the same methodology to the HG002 dataset, we determined the methylation status of genome-wide CGIs by summarizing the allelic methylation status of CpG sites contained in each CGI (Methods). We calculated the methylation score for each CGI as the average of the methylation scores of all CpGs comprising the CGI (Figure 3a). Next, we selected the 70 CGIs with the top 1% absolute differences (≥0.68) in methylation scores between the haploids of the diploid methylomes (Supplemental Table S1). For comparison, we analyzed the AK1 dataset and determined 139 CGIs (1.3%) had a methylation difference ≥0.68, which is consistent with the ratio in the HG002 dataset. Thus, we continued our analysis using HG002 data. We noted that the distances between these ASM CGIs and the PHVs were not necessarily small. Of the 70 ASM CGIs, 28 were separated by ≥500 bp and 9 by ≥1000 bp from their nearest PHV, which meant that the methylation status of the alleles of these CGIs could not be measured simultaneously when the reads were shorter than 500 or 1000 bp.

To confirm that the detected ASM CGIs are functionally relevant, we compared the ASM CGIs with genomic annotations from the combined segmentation by Segway and ChromHMM defined in the ENCODE project (Figure 3b) [34]. Of note, CGIs in general significantly overlapped the TSS, as expected. In contrast to this background, CGIs showing ASM overlapped with segments annotated as “transcribed regions” or “repressed regions” more than with the TSS. This result may seem somewhat contradictory, since any single gene cannot be both transcribed and repressed at the same time; however, this still may be a plausibly correct categorization for regions containing ASM genes because they can be, by definition, in two contrasting states in each of the alleles.

We also confirmed that our list of candidate ASM CGIs contains a number of CGIs overlapping with known imprinted genes. For example, we reproduced the expected ASM around known imprinted genes such as *MEST* (Figure 3c), *PEG13* (Figure 3d), *HYMAI*, and *ZNF597*, etc. (Supplemental Table S1) [35]. Indeed, CGIs with a larger methylation difference between two alleles were enriched with imprinted genes (p=0.007, U test). Thus, we again confirmed the validity of our method by successfully recovering the imprinted genes as ASM regions.

## 4. Discussion

In this work, we examined personal diploid methylomes to directly characterize the ASM status of genome-wide CGIs based on a set of PHVs specific to each sample. A crucial technical problem in this study was the accurate assignment of erroneous reads to haplotypes. The simulation revealed, however, that the accuracy would not be severely affected by sequencing errors, as they are random in nature [29]. On the other hand, wrong SNV calls/phasing can be a source of biased errors, which should be alleviated by using a PHV set of better quality. Therefore, the overall accuracy of detected methylation statuses would essentially replicate the prediction performance of the original methylation detection method, e.g., >90% for regions with sufficient sequencing depth, for example, 20-fold on each allele [17].

Compared with previous studies employing short read sequencing [12,13,16], one novelty of our approach is that we called methylation using kinetic information from long SMRT reads. We did not employ any chemical treatments, such as bisulfite conversion, which cleaves DNA into small fragments of <1500 bp [15]. By this design, we fully exploited the lengths of the PacBio reads (>8000 bp in our data). We determined the allelic origins of more than half of the sequencing data. Thus, we were able to cover more CpGs in the genome. We previously reported that a read coverage of ∼20-fold is required for detecting regional CpG methylation [17]. In cases in which sufficient read coverage is available for each allele after separation, this read coverage value can include a margin, since some reads will not contain any informative PHV and will be filtered out. Therefore, 40–50-fold of reads would be sufficient for the detection of ASM.

Compared with other existing methods, ours does not rely on the availability of restriction sites around the CpG sites, which makes our method potentially more comprehensive. Another important advantage of our method is that long reads enable the detection of ASM associated with distal heterozygous variants, and we demonstrated that such cases are not necessarily rare, as illustrated in Figure 2a,b.

As we demonstrated, comprehensive information about the genome-wide ASM status may complement ASE observations if we assume that the methylation status of promoter CGIs are correlated with transcriptional activity. We described a couple of examples to support this hypothesis using RNA-seq data in the AK1 dataset and indicated that the analysis of ASM could recapture some imprinted genes in the HG002 dataset. While we cannot use epigenetic observation as a complete surrogate for expression data, it can complement the ASE statuses of transcripts when they are more difficult to observe due to lack of exonic PHVs.

We also demonstrated that long reads are essential for the study of ASM, given the sparse distribution of heterozygous variants within individuals. Availability of haplotype-resolved variants data along the genomes was essential for the method presented, and the advent of linked-reads technology (10× GemCode/Chromium) enabled us to extract long-range (100 kbp) co-occurrences of DNA sequences for that purpose [24] and also for accuracy of the haplotype-phasing matters; with phasing errors, the sensitivity of our method might be undermined, or methylation statuses might be swapped between the two alleles. While we recommend to try such linked-read technology first as it is less labor-intensive and it can simply provide additional linking information to conventional short read sequencing data, several available methods for chromosome-scale haplotype reconstruction have been developed in recent years; there are novel protocols such as Strand-seq [36], and CPTv2-seq [37], and/or one can combine existing techniques such as Hi-C [38], optical mapping (Bionano) [39], and long/short read sequencing as well as linked-read [36,39]. Such technology renders it easier to sequence individual genomes in a manner that the majority of variants are haplotype-phased. The more accessible the haplotype-phased genomes become, the more reasonable it becomes to study the epigenome, being aware of the existence of two alleles.

## Supplementary Materials

The following are available at http://www.mdpi.com/2073-4425/9/9/460/s1, Table S1: Detected CpG islands with allele-specific methylation and associated genes.

## Abbreviations

The following abbreviations are used in this manuscript:

ASM	Allele-specific methylation
ASE	Allele-specific expresssion
SNP	Single nucleotide polymorphism
SNV	Single nucleotide variant
PHV	Phased heterozygous variant
IPD	Inter-pulse duration
CGI	CpG island
