# Supplementary material for: A Statistical Method for Observing Personal Diploid Methylomes and Transcriptomes with Single-Molecule Real-Time Sequencing
Source: Genes (Basel). 2018 Sep 19;9(9):460. doi: 10.3390/genes9090460 (PMC6162384; doi:10.3390/genes9090460)
Supplement: Supplementary file 1 [file genes-09-00460-s001.pdf]

genomic position	# CpGs	distance to hetSNVs (bp)	difference in methylation score	genes overlapping
chr7:100866136-100866524	32	1387	1.00	
chr19:40223180-40223523	38	842	1.00	*CNTD2*
chr2:127695525-127695872	42	144	1.00	
chr1:158177643-158178064	35	35	1.00	
chr6:156396937-156397413	41	4	1.00	
chr1:30807274-30807665	30	2801	1.00	
chr16:4752408-4752786	30	2394	1.00	*ZNF500*
chr1:3732730-3733110	36	605	1.00	*TP73 **
chr16:3443098-3443569	41	544	1.00	*ZNF597 **
chr3:105353685-105354118	32	129	1.00	
chr15:81118374-81118726	41	16	1.00	
chr19:53537558-53538603	100	474	0.99	*ZNF331 **
chr4:14862884-14863177	31	233	0.99	*LINC00504*
chr2:238139430-238139813	36	28	0.99	*KLHL30*
chr6:144007779-144008710	111	3253	0.98	*HYMAI **
chr7:130490898-130493270	184	901	0.98	*MEST **
chr13:20414868-20415475	55	1012	0.95	*CRYL1*
chr11:44957064-44957479	35	294	0.95	
chr19:56838629-56839102	36	37	0.95	*PEG3 **
chr17:21463876-21464280	38	514	0.95	
chr17:50781274-50781577	35	724	0.93	
chr19:46495771-46496033	30	818	0.92	*PNMAL2*
chr20:3164475-3165100	60	604	0.92	*LZTS3*
chr3:185282769-185283108	33	584	0.92	*MAP3K13*
chr7:76150310-76150704	39	389	0.92	
chr5:1594123-1594912	89	608	0.92	*SDHAP3*
chr2:80322453-80322673	30	261	0.91	*CTNNA2*
chr6:291948-292839	120	300	0.91	*DUSP22*
chr7:158203950-158204335	42	360	0.90	*PTPRN2 **
chr8:38650781-38651230	52	103	0.90	
chr17:78040904-78041242	31	2690	0.89	*TNRC6C*
chr5:156850291-156850674	45	584	0.89	*PPP1R2P3*
chr2:25204232-25204708	57	395	0.88	*LINC01381*
chr8:140097738-140100885	210	580	0.88	*TRAPPC9(PEG13) **
chr9:21970914-21971191	35	937	0.88	*CDKN2A*
chr14:68628298-68628690	40	263	0.87	*RAD51B*
chr1:211416335-211416879	71	151	0.87	*LINC00467*

chr7:64100041-64100498	51	141	0.87	
chr17:28306868-28307444	53	566	0.86	*KRT18P55*
chr1:16226831-16227165	37	77	0.86	
chr2:120349370-120349960	42	2003	0.85	*INHBB*
chr14:95360338-95360604	33	698	0.85	
chr7:154304879-154305196	37	342	0.85	*DPP6*
chr2:130213360-130213800	60	2796	0.84	
chr4:6008141-6008558	54	115	0.84	
chr10:86535448-86535835	37	209	0.83	
chr7:57404610-57405091	38	740	0.82	
chr20:9506464-9506827	32	260	0.80	*LAMP5-AS*
chr15:58864846-58865260	40	37	0.80	
chr10:42475747-42476386	50	380	0.77	*LINC00839*
chr10:86262883-86263522	47	326	0.77	*GRID1*
chr2:121225750-121226164	31	708	0.76	
chr6:138795809-138796332	45	36	0.76	*ECT2L*
chr19:37403441-37403963	41	368	0.75	
chr22:36409984-36410592	48	31	0.74	
chr22:49168124-49168686	42	249	0.73	
chr20:3749714-3750434	66	901	0.73	*HSPB12*
chr4:4862729-4863107	40	264	0.71	*MSX1*
chr7:16586523-16586906	31	130	0.71	
chr3:126416207-126416588	42	605	0.70	*CFAP100*
chr4:169774838-169775123	30	384	0.70	
chr4:6658645-6659090	55	264	0.70	
chr1:2334568-2334993	30	1519	0.70	*MORN1*
chr7:31336231-31336928	69	390	0.70	*NEWROD6*
chr5:178165654-178165985	39	208	0.70	
chr9:36985989-36986927	99	129	0.69	*PAX5*
chr1:235642018-235642471	35	96	0.69	*GNG4*
chr15:96360082-96360482	35	57	0.69	
chr12:118981300-118982005	56	89	0.68	*SRRM4*
chr17:72640377-72640815	48	63	0.68	*LINC00511*

*** imprinted genes**
